# A putative effector UvHrip1 inhibits BAX-triggered cell death in *Nicotiana benthamiana*, and infection of *Ustilaginoidea virens* suppresses defense-related genes expression

**DOI:** 10.7717/peerj.9354

**Published:** 2020-06-12

**Authors:** Yingling Wang, Jing Li, Shibo Xiang, Jianming Zhou, Xunwen Peng, Yingfan Hai, Yan Wang, Shuai Li, Songhong Wei

**Affiliations:** College of Plant Protection, Department of Plant Pathology, Shenyang Agricultural University, Shenyang, Liaoning, China

**Keywords:** Effector, *Ustilaginoidea virens*, Innate immunity, Pathogenicity

## Abstract

Rice false smut (RFS), caused by *Ustilaginoidea virens*, is one of the most detrimental rice fungal diseases and pose a severe threat to rice production and quality. Effectors in *U. virens* often act as a set of essential virulence factors that play crucial roles in the interaction between host and the pathogen. Thus, the functions of each effector in *U. virens* need to be further explored. Here, we performed multiple alignment analysis and demonstrated a small secreted hypersensitive response-inducing protein (hrip), named UvHrip1, was highly conserved in fungi. The predicted SP of UvHrip1 was functional, which guided SUC secreted from yeast and was recognized by plant cells. The localization of UvHrip1 was mainly in the nucleus and cytoplasm monitored through the GFP fusion protein in *Nicotiana benthamiana* cells. *uvhrip1* was drastically up-regulated in the susceptible cultivar LYP9 of rice during the pathogen infection, while did not in the resistant cultivar IR28. We also proved that UvHrip1 suppressed the mammalian BAX-induced necrosis-like defense symptoms in *N. benthamiana*. Furthermore, patterns of expression of defense-related genes, *OsPR1#012* and *OsPR10b*, were regulated over *U. virens* infection in rice. Collectively, our data demonstrated that infection of *U. virens* suppresses defense-related genes expression and UvHrip1 was most likely a core effector in regulating plant immunity.

## Introduction

Rice false smut (RFS) caused by the ascomycetous fungus *Ustilaginoidea virens* (Cooke) Takah (teleomorph *Villosiclava virens*) is one of the most harmful fungal diseases in rice (Zhang et al., 2014; Fan et al., 2016; Tang et al., 2019). With heavy losses of rice production worldwide, RFS control methods have growing attention recently. *U. virens* infects the rice florets and forms false smut balls, which is covered by chlamydospore on the infected spikelets, thereby causing a significant yield loss of up to 50% around the world (Tang et al., 2013; Zheng et al., 2017). The false smut balls also contain a variety of mycotoxins, such as ustilaginoidins and ustiloxins. Twenty-six ustilaginoidins derivatives and seven ustiloxins have been isolated and identified so far. Previous reports indicated that these secondary metabolites inhibit the assembly of tubulin and mitosis of cells in eukaryotes, and are toxic to animals and humans. (Koyama et al., 1988; Luduena et al., 1994; Shan et al., 2012; Wang et al., 2016; Fu et al., 2017).

When a pathogen and host plant come in contact with each other several elicitors are released by the pathogen, as well as plant defense mechanisms are activated to combat the infection (Liu et al., 2014; Wang et al., 2018). Pathogen-associated molecules pattern (PAMP)from the pahthogen is recognized by the pathogen recognition receptor (PRR) of plant cells, and then active defense signals and trigger the PAMP-triggered immunity (PTI) (Macho & Zipfel, 2014). Adapted pathogens secrete a vast array of effectors into the plant cell to hijack the plant’s immune system (Dou & Zhou, 2012). Evolutionarily, plant cells have acquired R (resistance) genes that express R proteins, which detects and recognizes pathogen effectors specifically. Such interaction triggers rapid and robust defense responsesas hypersensitive response (HR), called effector-triggered immunity (ETI) (Jones & Dangl, 2006; Stergiopoulos & De Wit, 2009; Irieda et al., 2019).

Effectors of plant pathogens were found to regulate plant immunity signaling by different strategies (Lo et al., 2015). For example, SCRE2 in *U. virens* significantly inhibits PAMP triggered defense responds as gene expression and oxidative burst, and contributes to full virulence of *U. virens* to rice (Fang et al., 2019). Slp1 and Ecp6, secreted by *Magnaporthe oryzae* and *Cladosporium fulvum*, respectively, competitively binds chitin with the host chitin receptors CEBiP and OsCERK, thereby drastically perturbing the host immune response triggered by chitin and promoting fungal infection (De Jonge et al., 2010; Mentlak et al., 2012). Pit2 in *Ustilago maydis* suppresses the activity of apoplastic cysteine proteases (CP2) of maize, and the *pit2* knockout mutant was significantly attenuated in *U. maydis* virulence to host (Mueller et al., 2013). The core effector Pep1 suppresses peroxidase POX12-drived oxidative burst and promote the infection of *U. maydis* in maize (Hemetsberger et al., 2012; Hemetsberger et al., 2015). A lipase domain-containing protein FGL1 suppresses the activity of callose synthase via releasing free fatty acids, decreases callose formation during *Fusarium graminearum* infection and thus plays an essential role in *F. graminearum* virulence (Blumke et al., 2014). Furthermore, the effectors LysM and AGLIP1, secreted by necrotrophic pathogen *Rhizoctonia solani*, inhibit chitin-induced immunity and promote pathogen infection to host (Dolfors et al., 2019; Li et al., 2019).

Plant cell-death symptoms triggered by the mouse pro-apoptotic protein BAX are physiologically similar to ETI triggered hypersensitive response. Testing the ability of inhibiting BAX-induced cell death has been a useful method for the pathogen effectors immunosuppressive ability (Lacomme & Santa, 1999; Chen et al., 2018). In *Phytophthora sojae*, most avirulence homolog (Avh) effectors which contain RXLR-dEER motifs are identified to inhibit BAX-induced cell death in *Nicotiana benthamiana* (Wang et al., 2011). *Heterodera avenae* secreted a variety of effectors, including most members of G16B09-like effector protein family, suppress cell death triggered by BAX in *N. benthamiana* (Chen et al., 2018; Yang et al., 2019). Besides, SCREs, UvBI-1 in *U. virens* and Pst_8713 in *Puccinia striiformis* f. sp. *tritici* significantly suppresses BAX-triggered cell death in *N. benthamiana*, and play an essential role to the pathogen virulence, respectively (Zhao et al., 2018; Fang et al., 2019; Xie et al., 2019; Zhang et al., 2020).

With the help of the recently-discovered genome, the molecular mechanism of *U. virens* pathogenicity has been further evaluated. *U. virens* encodes at least 628 potential secreted proteins, 193 of them, are relatively small (<400 amino acids) and cysteine-rich (≥4), which are thought to be hypothetical effectors. The cell death inhibition assays in *N. benthamiana* leaves, and the transcriptome analysis at different periods after pathogen infection, suggest that most effectors could manipulate the plant immune responses and promote the successful colonization of pathogens in the host (Zhang et al., 2014). Furthermore, many hypothetical effectors induce defense responses both in host rice and non-host *N. benthamiana*, and the signal peptides of these proteins are critical to their ability to cause cell death (Fang et al., 2016). Collectively, many hypothetical effectors can afffect plant immunity and play a key role in *U. virens* infection. However, the functions of most effector proteins are still unknown and need to further explore.

In this study, we found a putative secreted protein named UvHrip1 (protein ID: UVI_02019870) was conserved in fungi. We first ascertained UvHrip1 acts as an effector through yeast secretion, cell translocation, together with differential expression analysis assays. We further proved that UvHrip1 inhibits BAX-triggered cell death in *N. benthamiana*. Patterns of expression of defense-related genes, *OsPR1#012* and *OsPR10b*, were regulated over *U. virens* infection in rice. Taken together, UvHrip1 was demonstrated to be a core effector, which contributed to regulate plant immunity.

## Materials & Methods

### Plant materials, pathogen strains and growth conditions

*U. virens* isolate strain P1 was cultured using PSA medium (200 g peeled potato extract boiled in water, 20 g sucrose and 16 g agar/L). *N. benthamiana* was growth in an artificial climate chamber at 14 h light (25 °C)/12 h dark (23 °C). *Agrobacterium* GV3101 and EHA105 for transient expression were cultured using LB medium (10 g tryptone, 5 g yeast extract and 10 g NaCl/L). Yeast strains YTK12 was cultured using YPDA medium (10 g yeast extract, 20 g peptone, 20 g glucose, 0.03 g adenine hemisulfate/L). In this study, the concentrations of antibiotics were used as follows (µg/ml): rifampin, 25; kanamycin, 50. All data were repeated at least three times, and the results were similar. Strains and plasmids involved in the study were shown in Table S1.

### Plasmids construction

The total RNA of *U. virens* was extracted following the RNA extraction kit (TaKaRa), and the concentration and quality of that were determined by NanoDrop 2000. Complementary DNA (cDNA) synthesis was carried out via PrimeScript™ 1st Strand cDNA Synthesis Kit (TaKaRa). The full-length and the truncated without signal peptide of UvHrip1 coding sequence amplified by *Phanta* Max ultra-fidelity DNA polymerase using the cDNA as a template.

For BAX-induced cell death inhibition and apoplastic protein detecting assay, PCR products were digested by *Xma* I and *Sal* I and subcloned into pGR107 (Jones et al., 1999). For testing the signal peptide function of UvHrip1, the DNA fragment coding the first 17 amino acid residues of UvHrip1 was cloned into pSUC2T7M13ORI (pSUC2) vector (Oh et al., 2009). For subcellular localization, the PCR products containing the coding sequence of UvHrip1 and UvHrip1^NSP^, was cloned into pCAMBIA1301-35S-*gfp* and pGD-*gfp* (Goodin et al., 2002; Fang et al., 2019; Li et al., 2019) after digestion with *Sac* I/*Kpn* I and *Xho* I/*Bam*H I, respectively. All recombination constructs were determined by sequencing. Primers used in this study areshown in Table S2.

### Transient expression of proteins in *N. benthamiana* mediated by *Agrobacterium*

The constructed plasmid was transformed into *A. tumefaciens* strains GV3101 and EHA105, respectively, by the freeze-thaw method (Deblaere et al., 1985). The positive transformation was verified by PCR. The overnight cultured *Agrobacterium* carrying the correct plasmid was collected and resuspended in 10 mM MgCl_2_ buffer (containing 10 mM MES, 10 mM acetosyringone). The optical cell density was adjusted to OD600 = 0.5 for UvHrip1 or UvHrip1^NSP^-containing strain; OD600 = 0.3 for BAX-containing strain. The *Agrobacterium* containing the corresponding plasmid was infiltrated into 4–5 weeks old *N. benthamiana* by needleless syringe. Photos were taken after 2–3 days post-inoculation.

### Inoculation of *U. virens* in rice and quantitative real-time reverse transcription-polymerase chain reaction (qRT-PCR) assay

Artificial inoculation was performed as described previously (Fang et al., 2019). Briefly, P1 was cultured for 5–7 days at 120 rpm/min and 28 °C in the dark in PS medium. Mycelia and conidia were re-mixed at a concentration of 1 × 10^6^ conidia/ml with PS medium. Use a needle syringe to inject the inocula into the panicles before rice heading stage. Rice spikelets collected at 0, 24, 48, 72 and 96 h post-inoculation were stored at −80 °C for subsequent experiments.

RNA extraction and cDNA synthesis were performed as described above. qRT-PCR was performed by qPCR Master Mix from Vazyme Biotech Co., Ltd and detected by the Bio-Red CFX96 system. The internal reference gene primers used for normalizing each sample were listed in Table S2.

### Validation of UvHrip1 predicted signal peptide

Functional validation of UvHrip1 predicted SP was performed though yeast secretion assay (Jacobs et al., 1997). The 0.5 µg plasmids were transformed into YTK12, which is an invertase-deficient yeast strain, by using yeast transformation kit (Zymo Research). Positive transformants were grown on CMD-W medium (6.7 g yeast N base without amino acids, 0.75 g tryptophan dropout supplement, 20 g sucrose, 1 g glucose and 15 g agar / L). Invertase secretion was confirmed by the yeast colonies multiplied on YPRAA medium (10 g yeast extract, 20 g peptone, 20 g raffinose and antimycin A at 2 µg/L)

### Ion leakage in *N. benthamiana* leaf discs

The ion leakage assay in *N. benthamiana* leaf discs to evaluate cell death was as described previously (Fang et al., 2016).

### Isolating apoplastic protein from *N. benthamiana* leaves

The apoplastic protein was extracted from 4–5 weeks old *N. benthamiana* leaves with minor changes as previously published (Weinhold et al., 2015). Briefly, 5–6 leaves of 3 days after *Agrobacterium*-inoculated were harvested and incubated into optimized cold PBS solution (0.05 M Na_3_PO_4_, 0.3 M NaCl, pH 7.5), vacuum for around 20 min to immerse the leaves in the solution entirely. Samples were taken out, dried and transferred into a 50 ml syringe, which was put inside the 50 ml centrifuge tube. Then spin at 1,000 g for 5 min at 4 °C. The liquid in the tube is the apoplastic fluid. Transient expression of proteins in *N. benthamiana* was performed according to *Agrobacterium*-mediated transformation.

## Results

### UvHrip1 is highly conserved in fungi

**Figure 1:**
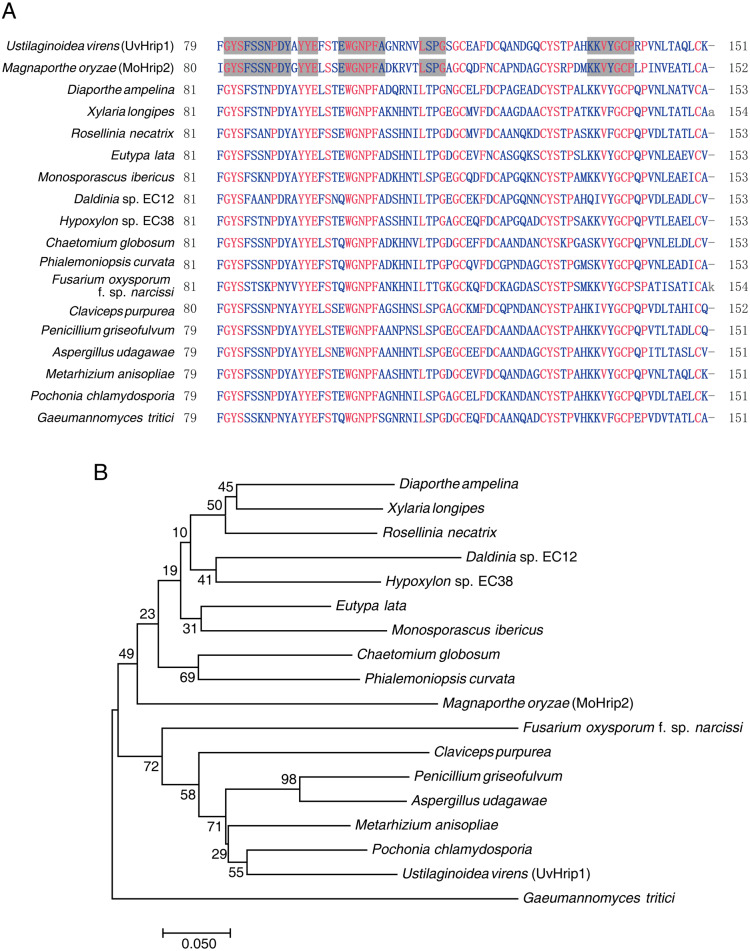
Conversation and similarity analysis of UvHrip1 with known fungal pathogen proteins. (A) Multiple alignment of UvHrip1 with other known 17 proteins. Highly and less conserved amino acids are clolored in red and blue, respectively. Grey shaded indicated highly conserved columns between *Ustilaginoidea virens* (UvHrip1) and *Magnaporthe oryzae* (MoHrip2). (B) Neighbor-joining tree analysis of UvHrip1 with other 17 proteins from various species. The MGEA version 7 was used for Neighbor-joining construction. 0.1 indicated the genetic distance and is shown by a scale bar in the lower left.

Pathogen core effector is very conserved in many plant pathogens (Li et al., 2019). Based on BLAST searches against the EMBL-EBI database (https://www.ebi.ac.uk/), UvHrip1 was found to encodes a small protein with 151 amino acid, and was predicted to be a hypersensitive response-inducing protein (hrip) elicitor. The sequence of UvHrip1 was highly identity with MoHrip2 identified in *M. oryzae* (Chen et al., 2014). For further explore sequence conservation of UvHrip1, multiple amino acid alignment analysis through the NCBI database was performed, and showed UvHrip1 is highly conserved in the known pathogenic fungi proteins (Fig. 1A). Furthermore, Neighbor-joining tree analysis also demonstrated that the homolog of UvHrip1 was widely present in pathogenic fungi, and the evolutionary relationship between the proteins from *U. virens* and *Pochonia chlamydosporia* was the closest (Fig. 1B).

### Functional validation of UvHrip1 predicted signal peptide (SP)

**Figure 2:**
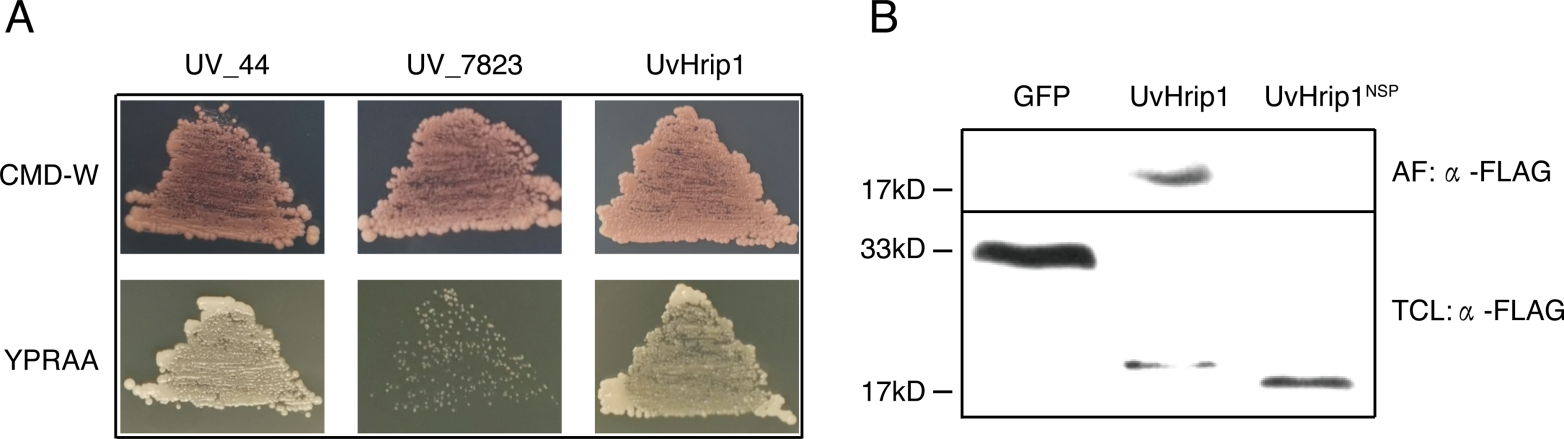
The signal peptide (SP) of UvHrip1 is functional. (A) SP of UvHrip1 is functional in yeast. CMD-W medium were used to select yeast strain YTK12 carrying the pSUC2 vector. YPRAA medium contains raffinose as sole carbon source was used to indicate invertase secretion. The predict SP sequences of UV_44 and UV_7823 in *Ustilaginoidea virens* were used as positive and negative controls, respectively. (B) UvHrip1-FLAG was detected in apoplastic fluid (AF) and total cell lysate (TCL) via Western blot analysis. Agroinfiltration sites of each *N. benthamiana* leaf expressing UvHrip1, UvHrip1^NSP^ and GFP, respectively. Samples were collected after 3 days post-inoculation of the apoplastic fluid of leaves. The proteins with a FLAG tag were detected by immunoblotting with an anti-FLAG antibody (*α*-FLAG).

UvHrip1 is a putative secreted protein that contains a predicted SP at the first 17 amino acid residues of N-terminal. In order to verify the function of the predicted SP, an invertase secretion assay was performed by previous study (Jacobs et al., 1997; Fang et al., 2016; Fan et al., 2019). The nucleotide sequence encoding the first 17 amino acid residues of UvHrip1 was cloned to the N-terminal of the SP-deleted *SUC2* gene. The SUC2 is an invertase, which hydrolyzes polysaccharides (such as sucrose and raffinose) into monosaccharides (such as glucose and fructose) to provide carbon source for yeast growth (Jacobs et al., 1997; Fang et al., 2016; Fan et al., 2019). The recombinant construct was transformed into YTK12, a SUC-deficient yeast strain, which cannot hydrolyzes raffinose as a carbon source. Recombinant SUC2 guided by bona fide SP can be secreted into YPRAA medium by YTK12, allowing the yeast to grow on the medium with raffinose as the only carbon source. As expected, the yeast YTK12 expressing SUC2 fused with the SP of UvHrip1 could grow on the YPRAA medium. The N-terminal peptide of UV_44 and UV_7823 act as positive and negative controls, respectively, were also cloned to the SP-deleted SUC2 and expressed as fusion proteins in YTK12 (Fang et al., 2016) (Fig. 2A).

To further investigate whether the SP of UvHrip1 could be recognized by plant cells, the recombinant vectors carrying *uvhrip1*, *uvhrip1*^NSP^ (the truncated without SP) and *gfp* were infiltrated into *N. benthamiana* by using *Agrobacterium*-mediated transformation, respectively. Apoplastic fluid was extracted from the inoculated leaves, and then detected by Western blot assay. The results showed that the full length with a FLAG tag at C-terminal of UvHrip1 was detected from the apoplastic protein, while UvHrip1^NSP^ and GFP did not (Fig. 2B), indicating the SP of UvHrip1 could be recognized by *N. benthamiana* cells.

These results demonstrated that the predicted SP of UvHrip1 is functional in mediating secretory pathway.

### UvHrip1 is mainly localized in the nucleus and cytoplasm

**Figure 3:**
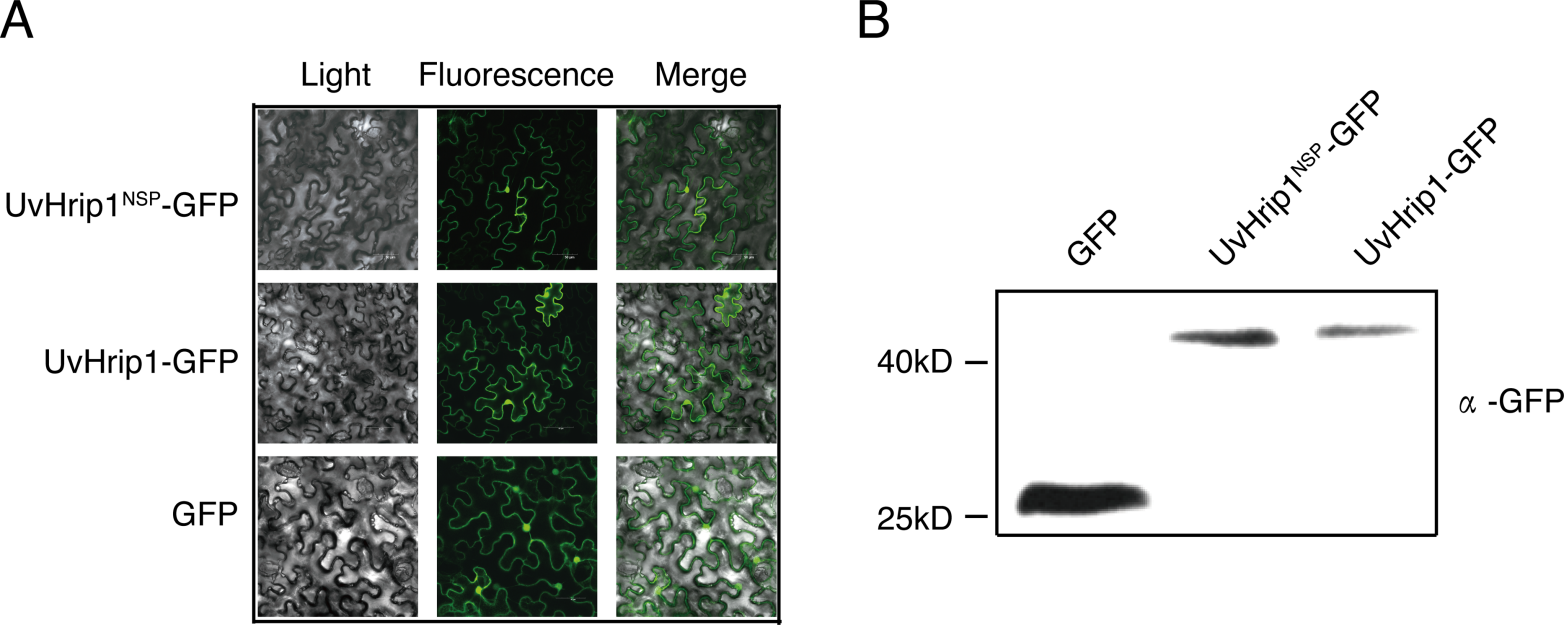
Subcellular localization of UvHrip1-GFP and UvHrip1^NSP^-GFP transiently expressed in *Nicotiana benthamiana*. (A) The green fluorescence of UvHrip1-GFP and UvHrip1^NSP^-GFP were detected in the nucleus and cytoplasm of *N. benthamiana* cells, respectively. The pCAMBIA1301-GFP construction was used as a control. The photos were taken under a laser scanning confocal microscopy 3 days after *Agrobacterium* inoculation. (B) UvHrip1-GFP and UvHrip1^NSP^-GFP were stably expressed in *N. benthamiana*. Agroinfiltration sites of each *N. benthamiana* leaf expressing UvHrip1, UvHrip1^NSP^ and GFP, respectively. Samples were collected from the infiltrated leaves after 3 days. The proteins with a GFP tag were detected by immunoblotting with an anti-GFP antibody (*α*-GFP).

To investigate the subcellular localization of UvHrip1 *in planta*. Nucleotide sequence encoding the full length and NSP of UvHrip1 was cloned in-frame with the N-terminal of *gfp*, respectively. The fusion protein and GFP were transiently expressed in *N. benthamiana* by using *Agrobacterium*-mediated expression, respectively. The result showed that green fluorescence of UvHrip1-GFP and UvHrip1^NSP^-GFP were detected in the nucleus and cytoplasm, which exhibited a similar subcellular localization of GFP transiently expressed in the infiltrated *N. benthamiana* cells (Fig. 3A). Similar fluorescence was monitored when GFP was fused to the N-terminal of UvHrip1 and UvHrip1^NSP^, respectively (Fig. S1). Western blot demonstrated that GFP did not be truncated or released from the fusion protein in *N. benthamiana* cells (Fig. 3B).

### Differential expression analysis of *uvhrip1* in young rice panicles during *U. virens* infection

**Figure 4:**
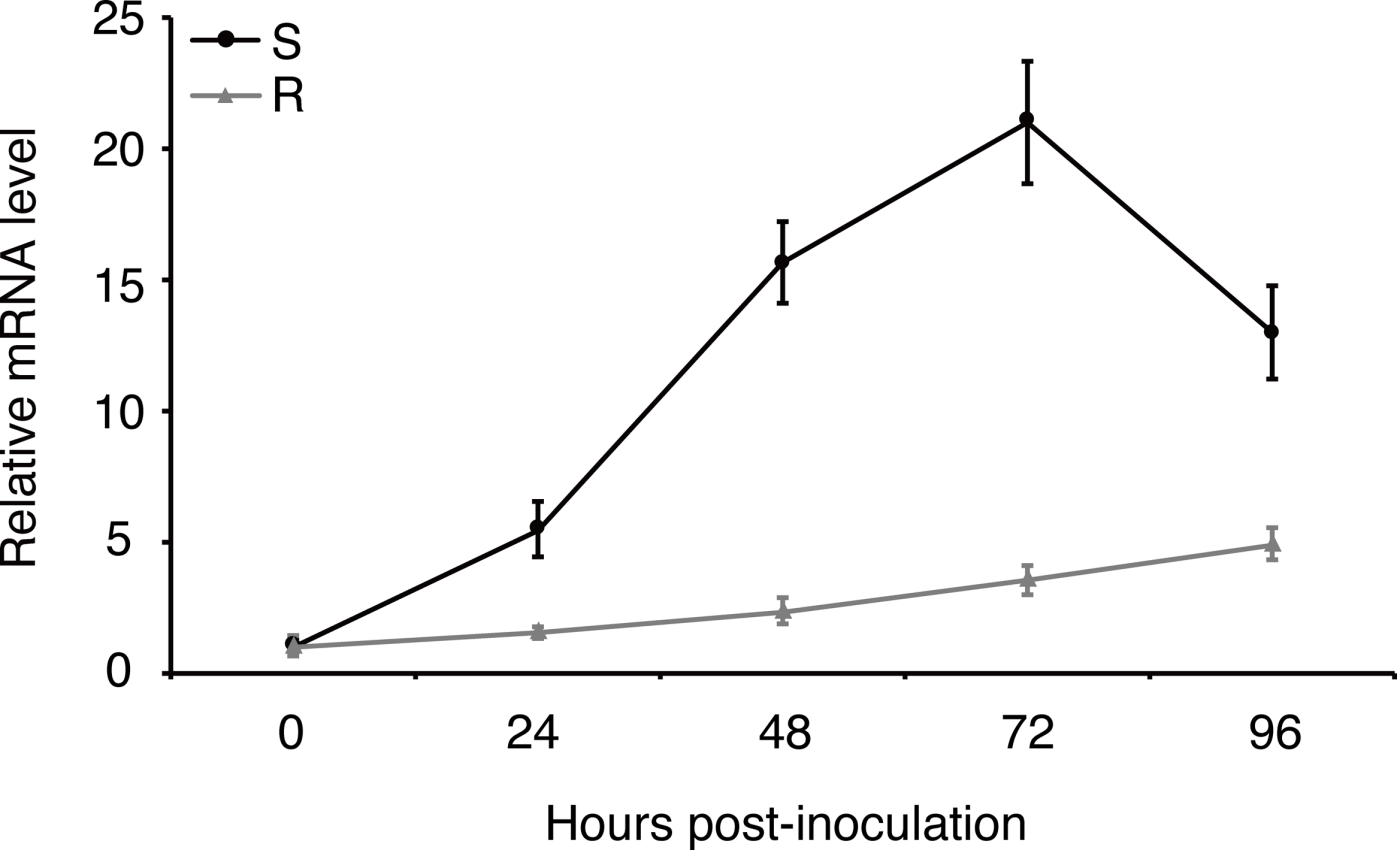
Differential expression analysis of *uvhrip1* during *Ustilaginoidea virens* infection of the P1-resistant and -susceptible rice cultivars. Gene expression was analysesed by quantitative real-time reverse transcription-polymerase chain reaction assay. Samples were collected from The *U. virens*-inoculated panicles of the P1-resistant cultivar IR28 (R) and susceptible cultivar LYP9 (S) at 0, 24, 48, 72 and 96 h post-inoculation. The reference gene *α*-*tubulin* normalized the gene expression level. Data are means ± standard error. The results shown are representative of three independent replicates with similar results.

Expression of effector genes are often transcriptionally regulated when filamentous plant pathogen infects to host (Li et al., 2019). In order to understand how *uvhrip1* expression is regulated during *U. virens* infection, a highly virulent strain P1 was artificially inoculated into young panicles of the rice resistant cultivar IR28 and susceptible cultivar LYP9, respectively. (Han et al., 2015; Fang et al., 2016). The expression level of *uvhrip1* was measured by qRT-PCR at 0, 24, 48, 72 and 96 h post-inoculation. Compared to the expression at 0 h post-inoculation, *uvhrip1* was transcriptionally induced throughout the period we detected, and was up-regulated approximately 20 fold at 72 h post-inoculated into the rice cultivar LYP9. By contrast, the expression level of *uvhrip1* was slightly induced after 72 h post-inoculated into the rice cultivar IR28 (Fig. 4). Therefore, the kinetics of *uvhrip1* expression indicated that UvHrip1 might be an effector that is beneficial to the virulence of *U. virens* during the rice infection.

### UvHrip1 inhibits BAX-induced cell death in *N. benthamiana*

**Figure 5:**
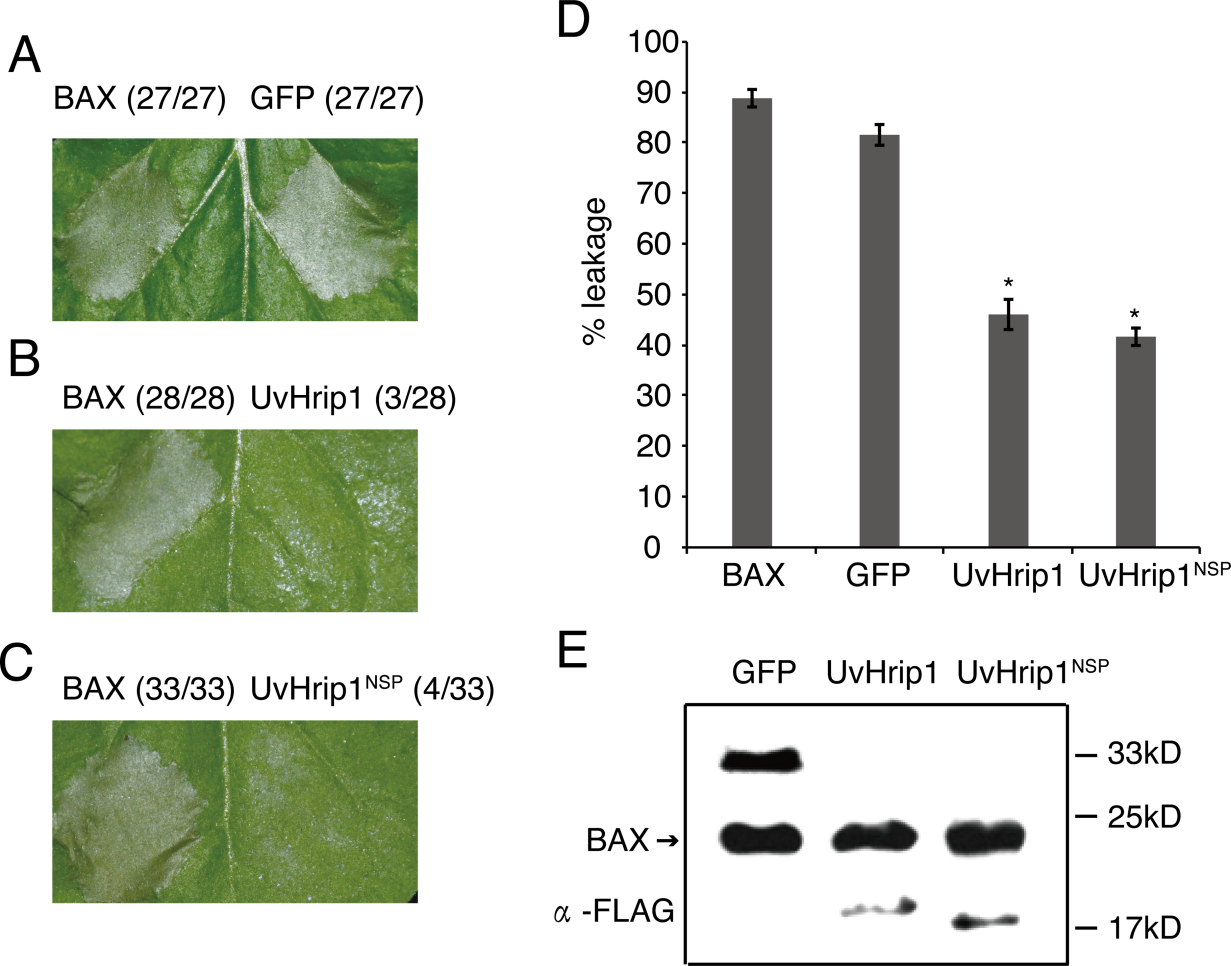
UvHrip1 suppresses BAX-triggered cell death in *Nicotiana benthamiana*. (A–C) Transiently expressed either UvHrip1 or UvHrip1^NSP^ inhibited cell death induced by BAX in *N. benthamiana* leaves. Agroinfiltration sites of each *N. benthamiana* leaf expressing GFP (A), UvHrip1 (B) and UvHrip1^NSP^ (C) were challenged with *Agrobacterium* expressing BAX , respectively. Agroinfiltration sites of each *N. benthamiana* leaf expressing BAX alone. Photographs were taken 3 days after *Agrobacterium* inoculating. Numbers, e.g., 27/27, indicate that 27 of 27 infiltrated leaves exhibited cell death phenotypes. (D) Ion leakage was measured from the induced cell death *N. benthamiana* leaves. Samples were collected from different Agroinfiltration sites of each *N. benthamiana* leaf expressing GFP, UvHrip1 and UvHrip1^NSP^ 4 days post-inolculation. The GFP construct was infiltrated as control. Data are means ± standard error (SE) from three independent experiments. Asterisks (*) indicate *P*-value <0.05, according to Student’s *t*-test. (E). BAX was stably expressed in *N. benthamiana*. Agroinfiltration sites of each *N. benthamiana* leaf expressing UvHrip1, UvHrip1^NSP^ and GFP, respectively. Samples were collected from the infiltrated leaves after 3 days. The proteins with a FLAG tag were detected by immunoblotting with an anti-FLAG antibody (*α*-FLAG).

Testing the ability of suppressing BAX-induced cell death is a useful method to identify functional effectors (Cheng et al., 2017). To investigate whether UvHrip1 regulates plant innate immunity, *Agrobacterium* strains carrying UvHrip1 and BAX were co-infiltrated into *N. benthamiana* leaves. UvHrip1 suppresses the BAX triggered cell death symptom in the infiltrated leaves, while GFP cannot. In addition, transiently expressed UvHrip1^NSP^ could also inhibit BAX mediated cell death in *N. benthamiana* leaves (Fig. 5A–C). Furthermore, ion leakage assay to correlate with cell death positively. The results showed the ion leakage of the leaves significantly reduced when co-expressing either UvHrip1 or UvHrip1^NSP^ with BAX comparison with that co-expressing GFP and BAX (Fig. 5D). The expression level of BAX was not altered when co-expressed with UvHrip1, UvHrip1^NSP^ or GFP in *N. benthamiana* leaves (Fig. 5E). These data demonstrated that UvHrip1 defense-related responses in *N. benthamiana*.

### Expression analysis of defense-related genes in young rice panicles during *U. virens* infection

**Figure 6:**
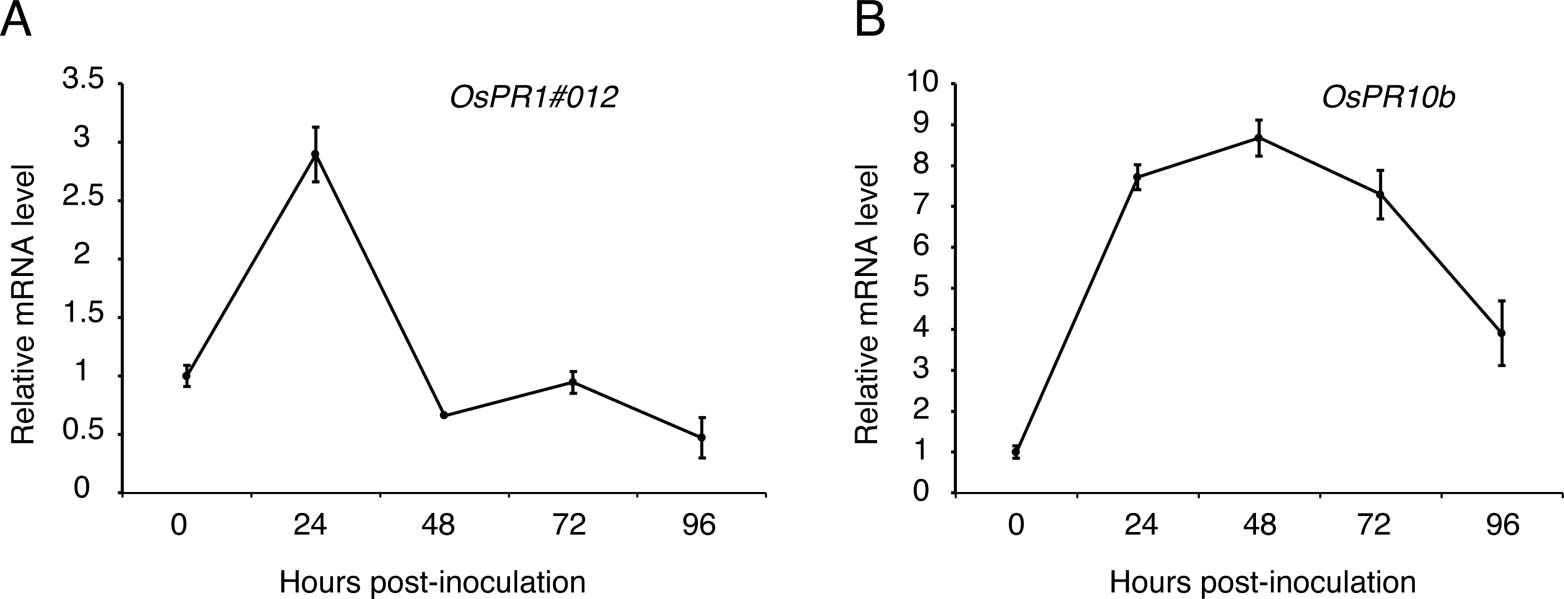
Expression analysis of defense-related genes during *Ustilaginoidea virens* infection to the rice cultivar LYP9. The rice spikelets for RNA preparation were collected at different time points (0, 24, 48, 72 and 96 h after P1 inoculation). *OsPR1#012* (A) and *OsPR10b* (B) expression was detected by qRT-PCR assay. The gene expression level was normalized by the reference gene *Os Actin*. Data are means ± standard error. The results shown are representative of three independent replicates with similar results.

To figure out whether the expression patterns of defense genes were regulated over P1 infection in young rice panicles, the expressions level of *OsPR1#012* and *OsPR10b* (Fan et al., 2015; Fan et al., 2019) were detected by qRT-PCR at 0, 24, 48, 72 and 96 h post-inoculation to the cultivar LYP9. The results showed that the expression of *OsPR1#012* was observably low at 48, 72 and 96 h post-inoculation (Fig. 6A). By contrast, *OsPR10b* was up-regulated at 24, 48 and 72 h post-inoculation, but inhibited at later time points (Fig. 6B). These results indicated that *U. virens* suppressed host defense-related gene expression when infected.

## Discussion

Rice false smut, caused by *U. virens*, occurs at the late stage of rice development, reduces grain yield and quality. The disease has been reported in most rice-growing areas of China and emerged as one of the major diseases in rice (Tang et al., 2013; Fan et al., 2016). Many studies have been carried out to reduce the yield loss caused by RFS. However, little is known about the molecular mechanism underlying the interaction between rice and *U. virens*. Phytopathogenic microbes secrete the majority of effectors to regulate plant immunity by targeting different host key components (Giraldo & Valent, 2013; Lo et al., 2015). More than 600 secreted proteins have been predicted in *U. virens* genome, 193 of which are identified as candidate effectors. The genes encoding many putative effectors were identified as being transcriptionally induced during *U. virens* infection in rice via expression profiling analysis, indicating they may be associated with inhibiting defense-associated responses (Zhang et al., 2014). In this study, we demonstrated that UvHrip1 as an effector regulates defense signaling in *N. benthamiana*.

The core effector shows a similar sequence and conserved motif across species (Hemetsberger et al., 2015; Li et al., 2019). BLAST searches against the EMBL-EBI database indicated UvHrip1 is a hypersensitive response-inducing protein (hrip) elicitor, which is similar to MoHrip2 in *M. oryzae*. Multiple alignment analysis demonstrated that full length of UvHrip1 and MoHrip2 shows 67% identities, and the motif is highly conserved in the known pathogenic fungi proteins. Although the evolutionary relationship between UvHrip1 and MoHrip2 are not closely (Fig. 1). The Hrip-elicitors have been identified to improve plant resistance to pathogen, such as Hrip1 from *Alternaria tenuissima* (Kulye et al., 2012), PaNie from *Pythium aphanidermatum* (Veit et al., 2001), and MoHrip1 from *M. oryzae* (Chen et al., 2014). The defense responses are often accompanied by HR, ion influx, accumulation of NO and production ROS (Hammond-Kosack & Parker, 2003). However, we cannot observe cell death symptoms within 3 days after UvHirp1-expressing *Agrobacterium* inoculated into *N. benthamiana*. Possibly, UvHirp1 induces cell death in the later time after *Agrobacterium* inoculation, or perceived by specific R protein as avirulence protein to trigger HR in the host. Therefore, the precise function of UvHirp1 will be confirmed by further experiments in rice.

UvHrip1 was ascertained as an effector through expression analysis, cell translocation and SP functional verification assay (Fang et al., 2016). UvHrip1 was predicted to contain SP at the first 17 amino acid residues of N-terminal. The prediction was demonstrated through the assays as yeast secretion and immunoblotting of apoplastic fluid, in which the SP was functional to guide UvHrip1 out of plant and yeast cells (Fig. 2). Subcellular localization detected by confocal microscopy showed that UvHrip1-GFP and UvHrip1^NSP^-GFP were mainly localized to cytoplasm and nucleus when transiently expressed in *N. benthamiana*, respectively (Fig. 3). The results indicated UvHrip1 secreted by *U. virens* might have multiple functions in plant (Li et al., 2015). However, the multiple cellular sites localization of UvHrip1 cannot be ruled out because the fusion constructs is overexpressed in *N. benthamiana*. Hence, the precise localization of the protein *in planta* needs to further explore. The common characteristic of functional effectors is that genes are often transcriptionally regulated when pathogen infects to host (Stergiopoulos & De Wit, 2009; Li et al., 2019). Interestingly, our result showed *uvhrip1* was significantly up-regulated when isolate P1 was inoculated in the susceptible rice cultivar LYP9, but not when inoculated in the resistant cultivar IR28 (Fig. 4). This phenomenon may be due to a specific protein, which recognizes and inhibits the function of UvHrip1 in the resistant cultivar IR28. A similar result has been shown that the expression level of *UV_7115* and *UV_7842* varied with *U. virens* infection in different disease resistance of rice cultivars (Fang et al., 2016).

A variety of effectors secreted by plant pathogens are shown to suppress cell death in plants and be required for full virulence for infection. The ability to inhibit BAX-induced cell death has been used to identify many putative functional effectors employing *Agrobacterium*-mediated transient expression assay in *N. benthamiana* (Wang et al., 2011; Zhang et al., 2014; Chen et al., 2018). In this study, we demonstrated that UvHrip1 truncated without signal peptide suppresses cell death triggered by BAX in *N. benthamiana* (Fig. 5), indicating UvHrip1 may function as a cytoplasmic effector and act inside the cell. Similar results were found in PsCRN115 of *P. sojae* and SCREs of *U. virens* (Liu et al., 2011; Fang et al., 2019; Zhang et al., 2020). Moreover, the SP could be recognized and guided UvHrip1 to the apoplast (Fig. 2), but the protein could still inhibit cell death. It is possible that UvHrip1 was first secreted out to the apoplastic space and then translocate back into the plant cells. The cellular localization of UvHrip1-GFP could further support the point (Fig. 3). Further investigations, such as reactive oxygen species, callose deposition, host target protein, will be carried out to further explore the function of UvHrip1.

Pathogens, which successfully colonize host tissues/organs, should have the ability to hijack or evade host immunity (Boller & He, 2009). Here, we found the expression patterns of rice defense-related genes, *OsPR1#012* and *OsPR10b*, were regulated over *U. virens* infection (Fig. 5). *OsPR1#012* is homologous of *PR1* in *Arabidopsis*, which is associated with the salicylic acid (SA) signaling pathway (Fan et al., 2015). Rice genome encodes 12 PR1 members, all of which are transcriptionally induced during compatible and/or incompatible *M. oryzae* strains infection (Mitsuhara et al., 2008). Expression of *OsPR1#012* was suppressed, while *OsPR10b*, which is a marker gene of jasmonic acid (JA) signaling pathway (De Vleesschauwer, Gheysen & Hofte, 2013), was highly expressed at the early time of infection (Fig. 5), indicating the SA- and JA-mediated defense pathways in rice spikelets may play an essential role in the interaction between rice and *U. virens*.

## Conclusion

In summary, a novel secreted protein UvHrip1 was identified and characterized as a conserved effector which suppresses immunity in non-host plant. However, the precise molecular mechanism of UvHrip1’s role in the interaction between rice and *U. virens* remains to be further elucidated.

##  Supplemental Information

10.7717/peerj.9354/supp-1 Figure S1 Subcellular localization of GFP-UvHrip1 and GFP-UvHrip1^NSP^transiently expressed in *Nicotiana benthamiana* The green fluorescence of GFP-UvHrip1 and GFP-UvHrip1^NSP^ were detected in the nucleus and cytoplasm of *N. benthamiana* cells, respectively. The vector pGD carrying *gfp* was used as a control. The photos were taken under a laser scanning confocal microscopy 3 days after *Agrobacterium* inoculation.

10.7717/peerj.9354/supp-2 Table S1 Strains and plasmids used in this study

10.7717/peerj.9354/supp-3 Table S2 The designed primers used in this study

10.7717/peerj.9354/supp-4 File S1 Raw Data

## Additional Information and Declarations

### Data Availability

The following information was supplied regarding data availability: The raw data of Figs. 1, 4, 5 and 6 are available in File S1.
