# Supplementary figures and images for: A putative effector UvHrip1 inhibits BAX-triggered cell death in Nicotiana benthamiana, and infection of Ustilaginoidea virens suppresses defense-related genes expression
Source: PeerJ. 2020 Jun 12;8:e9354. doi: 10.7717/peerj.9354 (PMC7295024; doi:10.7717/peerj.9354)

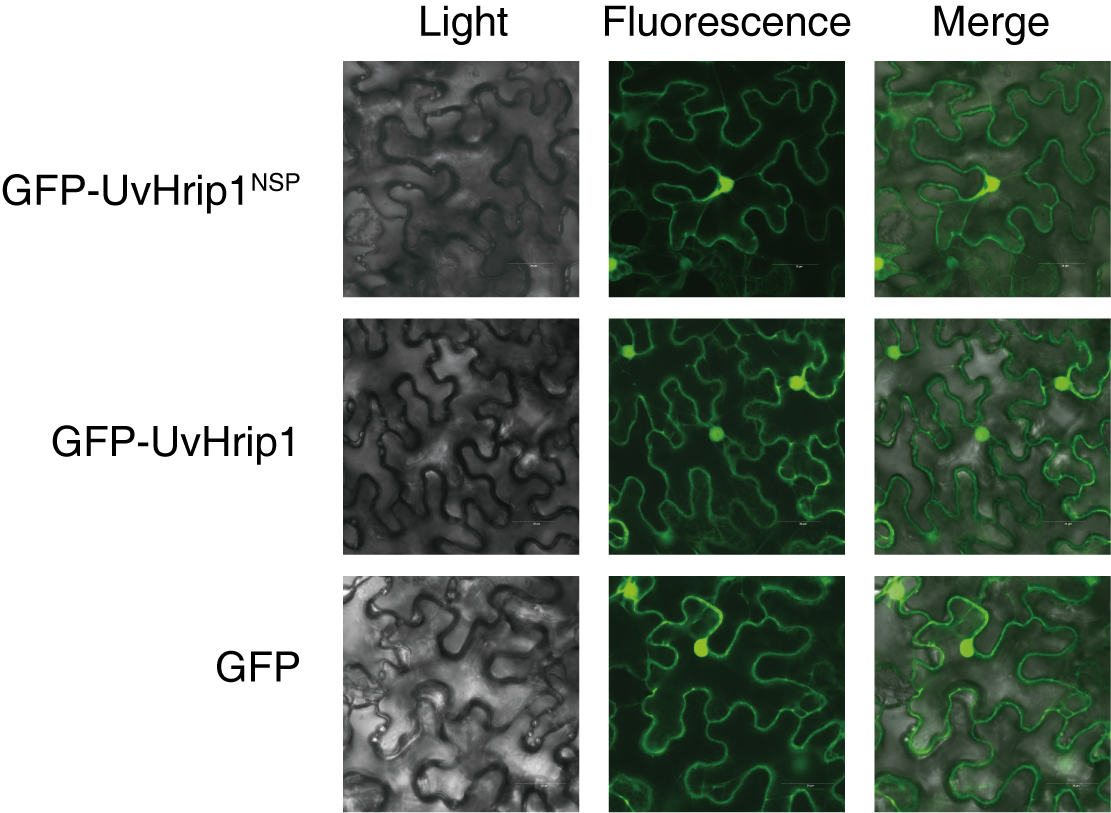

Supplement: Figure S1 — The green fluorescence of GFP-UvHrip1 and GFP-UvHrip1NSP were detected in the nucleus and cytoplasm of N. benthamiana cells, respectively. The vector pGD carrying gfp was used as a control. The photos were taken under a laser scanning confocal microscopy 3 days after Agrobacterium inoculation. [file peerj-08-9354-s001.png]

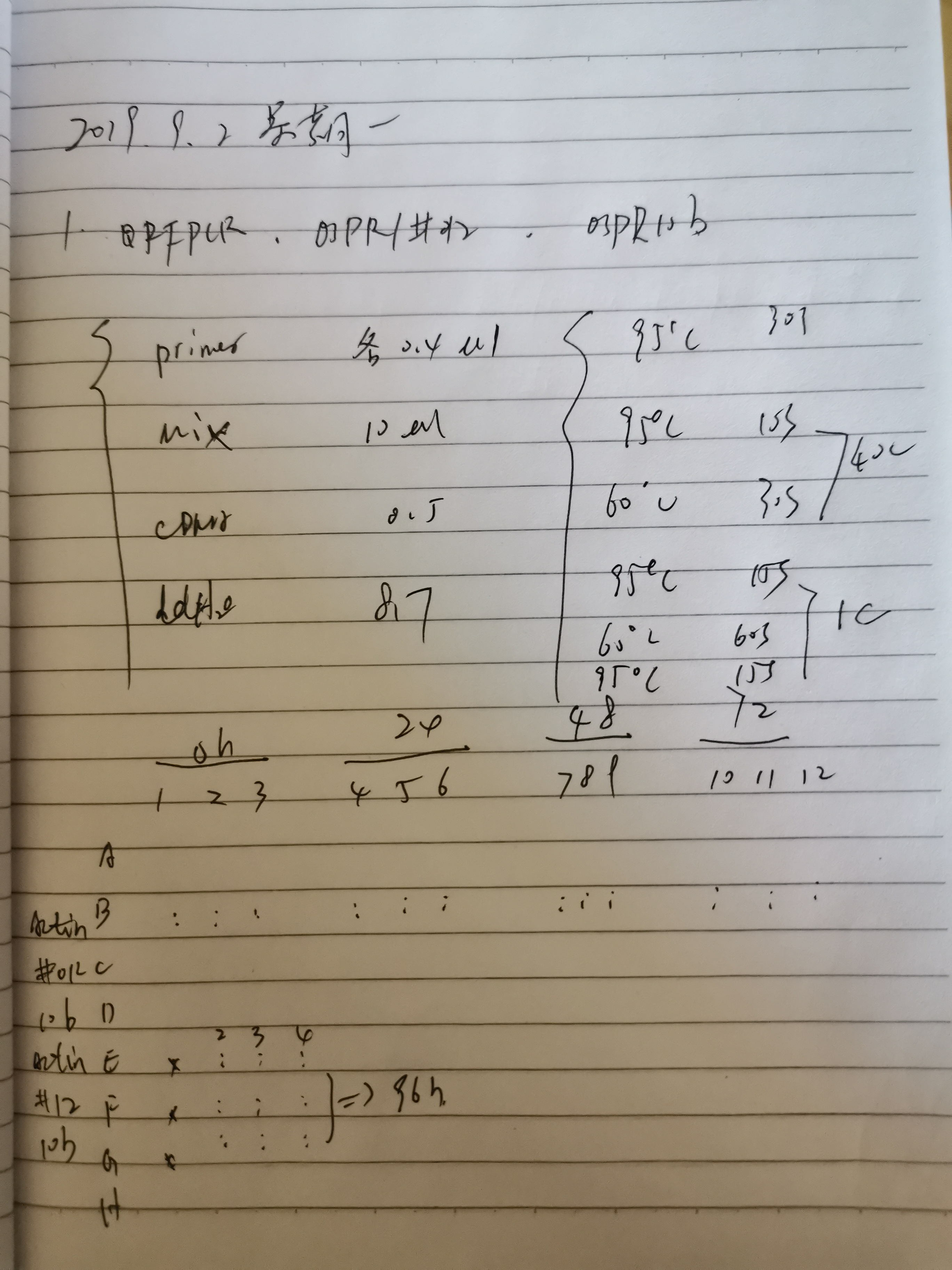

Supplement: File S1 [file peerj-08-9354-s004.zip › Raw Data/Fig. 5 Experimental information about qRT-PCR in the notebook.jpg]

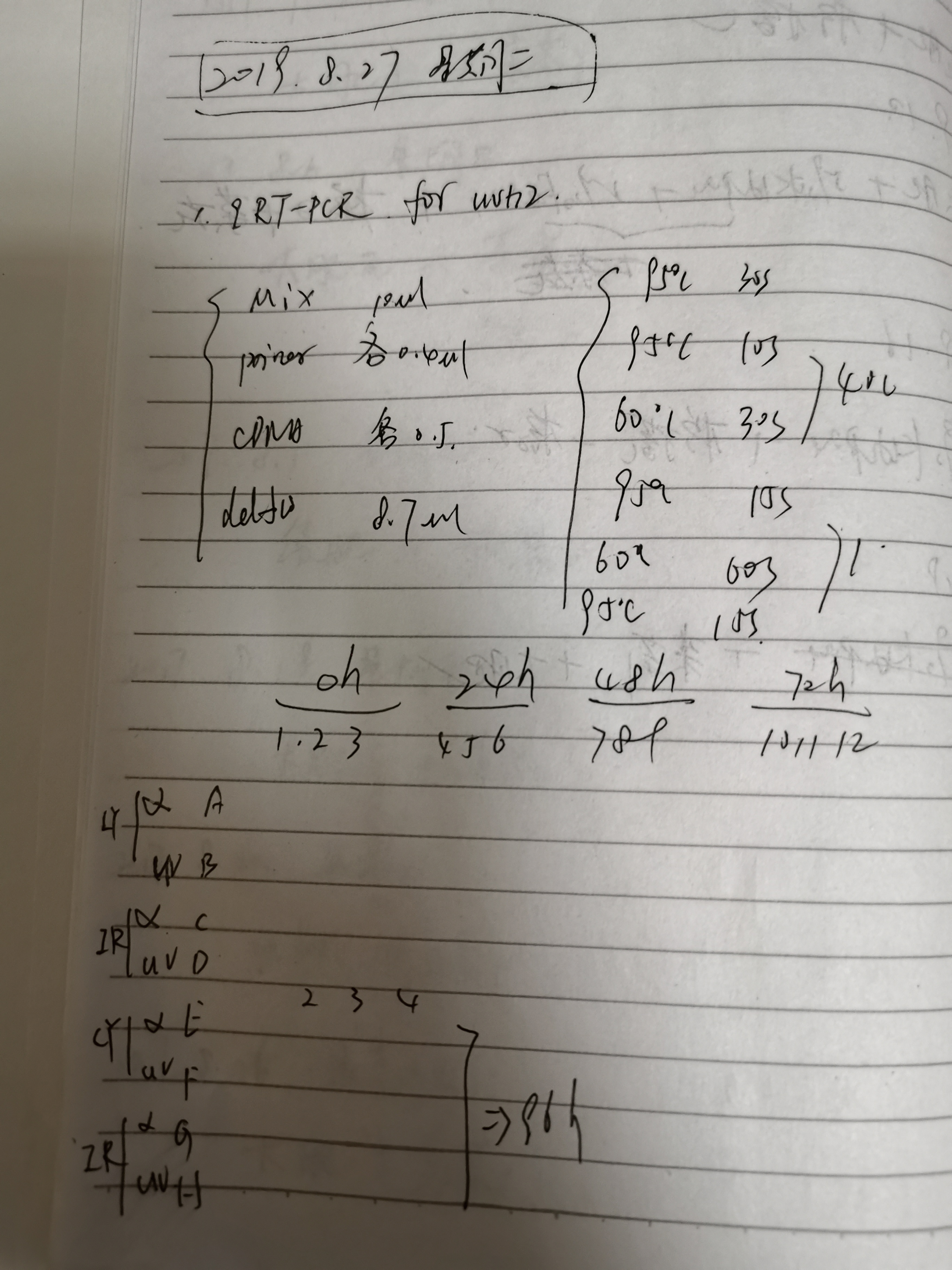

Supplement: File S1 [file peerj-08-9354-s004.zip › Raw Data/Fig. 4 Experimental information about qRT-PCR in the notebook.jpg]
