# Supplementary material for: A putative effector UvHrip1 inhibits BAX-triggered cell death in Nicotiana benthamiana, and infection of Ustilaginoidea virens suppresses defense-related genes expression
Source: PeerJ. 2020 Jun 12;8:e9354. doi: 10.7717/peerj.9354 (PMC7295024; doi:10.7717/peerj.9354)
Supplement: Table S1 [file peerj-08-9354-s002.docx]

Table S1**.** Strains and plasmids used in this study

| Strains/plasmids | Characteristics | References or source |
| --- | --- | --- |
| ***Escherichia coli*** |  |  |
| DH5a | High efficiency transformation | Lab collection |
| ***Agrobacterium*** |  |  |
| GV3101 | Wild-type, Rif^R*^ | Lab collection |
| EHA105 | Wild-type, Rif^R^ | Lab collection |
| **Yeast strain** |  |  |
| YTK12 | Truncated invertase gene | (*Fang et al., 2016*) |
| ***Ustilaginoidea virens*** |  |  |
| P1 | Wild type isolate | (*Han et al., 2015*) |
| **Plasmids** |  |  |
| pGR107 | Potato X virus（PVX）expression vector, Ka^R*^ | (*Jones et al., 1999*) |
| pGR107-*uvhrip1* |  | This study |
| pGR107- *uvhrip1^NSP^* |  | This study |
| pGR107-*bax* |  | This study |
| pGR107-*gfp* |  | This study |
| pSUC2 | Expression vectors for secretory function verification，Ka^R^ | (*Oh et al. 2009*) |
| pSUC2-*uvhrip1^sp^* |  | This study |
| pSUC2-*UV_44^SP^* |  | (*Fang et al., 2016*) |
| pSUC2-*UV_7823^SP^* |  | (*Fang et al., 2016*) |
| pCAMBIA1301-35S-*gfp* | Expression vector for subcellular localization，Ka^R^ | (*Li et al., 2019*) |
| pCAMBIA1301-35S- *uvhrip1*-*gfp* |  | This study |
| pCAMBIA1301-35S- *uvhrip1^NSP^*-*gfp* |  | This study |
| pGD-*gfp* | Expression vector for subcellular localization，Ka^R^ | (*Goodin et al., 2002; Fang et al., 2019*) |
| pGD-*gfp*-*uvhrip1* |  | This study |
| pGD-*gfp*-*uvhrip1^NSP^* |  | This study |

*Rif^R^ and Ka^R^ mean rifampin and kanamycin resistance, respectively.
