# Supplementary material for: A putative effector UvHrip1 inhibits BAX-triggered cell death in Nicotiana benthamiana, and infection of Ustilaginoidea virens suppresses defense-related genes expression
Source: PeerJ. 2020 Jun 12;8:e9354. doi: 10.7717/peerj.9354 (PMC7295024; doi:10.7717/peerj.9354)
Supplement: Table S2 [file peerj-08-9354-s003.docx]

**Table S2. The designed primers used in this study**

| Purpose of use | Primer name | DNA sequence |
| --- | --- | --- |
| BAX-inducing cell death inhibiting assay | UvHrip1-NSP-pGR107-XmaI-F | TAGTGGATCCCCCGGGCAGAACGCCGTCGTCA |
|  | UvHrip1-NSP-pGR107-SalI-R | TCATCGGCGGTCGACTTACTTGCAGAGCTGGGC |
|  | UvHrip1-pGR107-XmaI-F | TAGTGGATCCCCCGGGCAGAACGCCGTCGTCA |
|  | UvHrip1-pGR107-SalI-R | TCATCGGCGGTCGACTTACTTGCAGAGCTGGGC |
|  | BAX-pGR107-XmaI-F | TAGTGGATCCCCCGGGATGGACGGGTCCGGGG |
|  | BAX-pGR107-SalI-R | TCATCGGCGGTCGACTCAGCCCATCTTCTTCCAGA |
|  | GFP-pGR107-XmaI-F | TAGTGGATCCCCCGGGATGGTGAGCAAGGGCGAGG |
|  | GFP-pGR107-SalI-R | TCATCGGCGGTCGACTTACTTGTACAGCTCGTCCATGCC |
| Yeast secretion | UvHrip1^SP^-pSUC2-EcoR1-F | TTTAATTAAGAATTCATGAAGACCTCTGTTGTCGC |
|  | UvHrip1^SP^-pSUC2-Xho1-R | AGGGAGAACCTCGAGGTTGTACGGGAAAGACTGC |
| Subcellular localization | UvHrip1-NSP-pCAM1301-SacI-F | GAGAACACGGGGGACGAGCTCCAGAACGCCGTCGTCATCA |
|  | UvHrip1-NSP-pCAM1301-KpnI-R | TCTAGAGGATCCCCGGGTACCCTTGCAGAGCTGGGCAGT |
|  | UvHrip1-pCAM1301-SacI-F | GAGAACACGGGGGACGAGCTCATGAAGACCTCTGTTGTCG |
|  | UvHrip1-pCAM1301-KpnI-R | TCTAGAGGATCCCCGGGTACCCTTGCAGAGCTGGGCAGT |
|  | UvHrip1-NSP-pGD-XhoI-F | GTACAAGTCTCGAGCTATGCAGAACGCCGTCGTCA |
|  | UvHrip1-NSP-pGD-BamHI-R | AGATCCGGTGGATCCTTACTTGCAGAGCTGGGC |
|  | UvHrip1-pGD-XhoI-F | GTACAAGTCTCGAGCTATGAAGACCTCTGTTGTCGC |
|  | UvHrip1-pGD-BamHI-R | AGATCCGGTGGATCCTTACTTGCAGAGCTGGGC |
| Genes expression | UvHrip1-qPCR-F | ACAGGCCGTTGTTCTTTGGA |
|  | UvHrip1-qPCR-R | GGTTTCCGGCAAACGGATTC |
|  | OsPR1#012-qPCR-F | GCAGCTACGTTTACAAGCAGA |
|  | OsPR1#012-qPCR-R | GGCATCGGAGCAGTGAAACA |
|  | OsPR10b-qPCR-F | GTCGCGGTGTCGGTGGAGAG |
|  | OsPR10b-qPCR-R | ACGGCGTCGATGAATCCGGC |
|  | UV-α-tubulin-qPCR-F | GACAACTGCAACTCCCTCCA |
|  | UV-α-tubulin-qPCR-R | CTAGACGCTCCAGCATGAGG |
|  | OsActin-qPCR-F | TCCATCTTGGCATCTCTCAG |
|  | OsActin-qPCR-R | GTACCCGCATCAGGCATCTG |
